# *Scoliosis and Spinal Disorders* journal: a new, cutting-edge frontier in spine publishing

**DOI:** 10.1186/s13013-016-0061-3

**Published:** 2016-01-22

**Authors:** Theodoros B. Grivas, Dino Samartzis

**Affiliations:** Department of Orthopaedics and Traumatology, Tzaneio General Hospital of Piraeus, Tzani and Afendouli 1, Piraeus, 18536 Greece; Department of Orthopaedics and Traumatology, The University of Hong Kong, Pokfulam, Hong Kong, SAR China

With the start of 2016, we at the journal would like to wish all our readers, reviewers and Editorial Board members a most happy and prosperous new year. Ushering in this year, the journal has made some considerable changes. For one, the journal has been renamed *Scoliosis and Spinal Disorders*. We feel that with the ever-changing landscape of spinal conditions as well as health-care utilization and the need for a forum to voice cutting-edge and multidisciplinary spine research worldwide, [1, 2] we are broadening the scope of the journal to include all types of spinal deformities and disorders that entail conservative and surgical management that represent clinical or basic science research. The journal will continue to provide an integrated and balanced view of preventative, diagnostic, therapeutic, outcomes and cost-analyses research to enhance effective collaboration among spine specialists and scientists. To reflect the broad, multidisciplinary scope of the journal as well as its worldwide geographical presence, we will gradually revamp the Editorial Board. Thirdly, Dr. Dino Samartzis from the Department of Orthopaedics and Traumatology at The University of Hong Kong has been selected to serve as co-Editor-in-Chief alongside Dr. Theodoros B. Grivas. Dr. Samartzis has had a long and impactful career devoted to spine research, publishing and medical education throughout the world. His expertise and passion further reflects the journal’s commitment to quality, dedication to the broad spectrum of spinal disorders, multidisciplinary nature, and globalization of the spine community and research.

The start of a new year is also a time for reflection. In order to understand where we are headed, we must acknowledge and appreciate where we have been. After 2 years since the formation of the Society on Scoliosis Orthopaedic and Rehabilitation Treatment (SOSORT), the board of the society launched in 2006 the *Scoliosis* journal as its official journal and Dr. Hans-Rudolf Weiss became its first Editor-in-Chief [3]. Dr. Weiss worked tirelessly to organize and establish the journal as an open access publication by BioMed Central, a world leader in open access medical publications. During that period, the journal focused primarily on conservative management of scoliosis. In 2008, Dr. Grivas succeeded Dr. Weiss as the journal’s Editor-in-Chief after having served as an Associate Editor. Dr. Grivas helped align the journal with numerous other societies and expanded the editorial board substantially to include world renowned experts in the field. Under the leadership of Dr. Grivas, the journal further expanded its reach to include other spinal deformities besides scoliosis and also became receptive to articles addressing surgical management of spine deformities. Dr. Grivas also expanded the scope of the journal to address specialized thematic series, such as brace technology, rehabilitation schools for scoliosis, school screening for scoliosis, SOSORT consensus and guidelines, and the etiology of idiopathic scoliosis. The journal remains the official journal of SOSORT, but under the direction of Dr. Grivas the journal became further affiliated with numerous other spine societies in an effort to broaden its demographic and impact. As previously mentioned, the journal was subsequently renamed to its current title - *Scoliosis and Spinal Disorders*. Currently, the journal remains an open access, multidisciplinary, peer- reviewed publication that focuses on original, primary research articles as well as broad narrative/systematic reviews and meta-analyses. The journal is indexed in PubMed and in numerous other major search engines. BioMed Central remains the publisher of the journal.

Although the journal has enjoyed a rather auspicious start and sustainability, it has also managed to remain at the forefront of medical publishing. Some of the strengths of the journal are as follows:

- **Speed of publication** – The average time of article submission to acceptance is currently 42 days. We aim to have articles fully published and indexed within one month of acceptance.
- **Flexibility** – The journal has no limits to color images and number of authors for published articles. The journal also encourages deposition of datasets and other supplemental information to accompany the articles, such as multimedia. Articles are also provided links to PubMed and other relevant articles.
- **Full disclosure** – The journal firmly believes in full disclosure and transparency. As such, in addition to the authors’ disclosure statements, reviewer comments will also be available online to accompany the published article.
- **Worldwide accessibility and open access** – All published articles are available for full download without any fees to the reader or institution.
- **Indexing** – All published articles will be automatically indexed in PubMed, and in over 40 other abstracting and indexing databases.
- **Optimized reach** – The BioMed Central website has over 9 million visitors each month. In addition, a dedicated marketing and public relations team exists to help disseminate the published material within and beyond the academic community. Various tools (e.g., press releases, newsletters, social media, face-to-face reader meetings) will be utilized to disseminate the research findings in a timely manner and to maximize impact.
- **Article metrics** – The journal will track the number of times the article has been accessed, cited, covered in the media and blogs, shared via social media (e.g., Facebook, Twitter), and bookmarked/rated/discussed via bibliographic tools/sites (e.g., Papers, F1000, Mendeley). This service will further give both the authors and readers an indication of the global impact of the article.
- **Personalized approach** – The Editors-in-Chief and Editorial Board will provide a personalized and rapid attention to the needs and concerns of the authors. The Editors strive to perform thorough, fair and constructive peer review of all manuscripts, and in line with guidelines and best practice recommendations issued by organizations such as the Committee of Publication Ethics (COPE), and journal policies [4].

We invite you to join us in embarking in a new era for the journal – a new frontier in the review, publication and dissemination of research and knowledge of spine disorders and conditions. These new beginnings will entail a multidisciplinary approach to spine research, a globalization of broad spine topics, rigorous peer-review, expeditious publishing and continued open access, utilization of social media and other platforms to maximize reach and impact, and quality-driven evidence-based spine care in scientific reporting.
